# Immunological, Inflammatory, and Microbiota Determinants of Carpal Tunnel Syndrome: Evidence from Mendelian Randomization

**DOI:** 10.2174/0118715303452467260416073725

**Published:** 2026-04-30

**Authors:** Zeyu Zhou, Shuwei Liang, Zenghui Liu, Aoni Wu, Qiyu Sun

**Affiliations:** 1 Department of Clinical Laboratory, Affiliated Hospital of Chengde Medical College, Chengde, Hebei Province, China;; 2 Hebei Key Laboratory of Panvascular Diseases, Chengde, Hebei Province, China;; 3 Department of Immunology, Mudanjiang Medical University, Mudanjiang, Heilongjiang, China

**Keywords:** Immune cells, inflammatory proteins, gut microbiota, skin microbiota, Mendelian randomization, carpal tunnel syndrome

## Abstract

**Introduction:**

Carpal Tunnel Syndrome (CTS) is a common peripheral neuropathy, and immune dysregulation and microbial dysbiosis are believed to play a role in its development. However, the cause-and-effect relationships have yet to be clarified.

**Methods:**

Using publicly available Genome-Wide Association Study (GWAS), there are 731 immune cell phenotypes, 91 inflammatory proteins, 150 skin microbiota taxon, and 473 gut microbiota taxon based on two-sample Mendelian Randomization (MR) analysis to test whether there is a causal relationship between them and CTS. The results from the study were shown to have some degree of stability as demonstrated by various sensitivity analyses, which included running heterogeneity tests, performing MR -PRESSO, and running MR-Egger regressions. On the other hand, reverse MR was performed to verify the direction of the association. In addition, a two-step MR mediation analysis was conducted to explore whether there was a mediation effect of gut microbiota and skin microbiota, respectively, of immune and inflammatory traits on CTS.

**Results:**

22 Immune cell traits, 4 Inflammatory proteins, 18 gut microbiota taxa, and 3 skin microbiota taxa are causally associated with CTS. Reverse MR suggested feedback effects of CTS on select immune traits and gut microbiota. Mediation analysis revealed 4 gut microbiota taxa that substantially mediated immune/inflammatory effects upon CTS, with mediation rates as high as 44%; however, skin microbiota did not demonstrate any mediation.

**Discussion:**

The immune dysregulation, inflammation, and the gut microbiota that cause CTS are all revealed through this research. Mendelian randomization analysis suggests that traits and inflammatory proteins of immune cells directly increase the risk of CTS, and certain types of gut microbes partially mediate these effects. Therefore, the results show a central role of the immune-gut axis in CTS pathogenesis, and suggest a systemic, rather than a local, immune-microbial interaction in disease development.

**Conclusion:**

We provided the first causal evidence that immune cells, inflammatory proteins, and CTS risk are causally associated with some specific taxa of gut microbiota. This contributes to a better understanding of the immune-microbiome interactions in the process of occurrence and development of CTS, and also provides theoretical support for precision prevention and treatment.

## 1 INTRODUCTION

Carpal Tunnel Syndrome (CTS) is one of the most common peripheral neuropathies in the upper limb, and CTS mainly presents with pain, numbness, and paraesthesia of the median nerve territory [1]. The typical symptoms are hand numbness, sensory disturbance, pain, weakness, and reduced quality of life, which are prone to aggravation at night and greatly affect daily life and work [2-4]. Epidemiological data show that CTS has a high global incidence, with an annual incidence rate of about 3% to 6% in the general population [5], and its incidence is on the rise. Identifying risk factors for CTS can be used to prevent and accurately treat it. Besides mechanical compression, CTS has been linked to multiple systemic diseases and metabolic disorders, including aging, diabetes mellitus, rheumatoid arthritis, hypothyroidism, and chronic kidney disease (particularly in patients on dialysis with distal radius fracture) [6].

In recent years, accumulating evidence indicates that there are immune system abnormalities in the pathogenesis of CTS. The immune cells, such as T cells (CD3^+^cells), are more likely to infiltrate into the lesion site of CTS, and the “non-inflammatory fibrosis” was regarded as the only pathological basis [7]. In the experimental nerve injury model, peripheral immune cells migrate to injured nerves, DRGs, and the spinal cord area, and trigger neuronal hyperexcitability and permanent pain, which provides possible mechanistic insight into neuropathic pain in CTS [8]. In addition, low-grade systemic inflammation has been associated with CTS progression; for example, high CRP levels are significantly associated with proximal symptom spread, suggesting that CRP and other inflammatory markers may mediate the extension of disease [9]. Some cytokines, such as TGF-β1 and MIP-1β, and their corresponding gene polymorphisms may also have an effect on CTS susceptibility and course [10, 11].

In addition to immune and inflammatory mechanisms, the microbiota has also recently emerged as a possible participant in CTS, which includes both gut and skin microbiota. Gut microbiota regulates the host immunity and inflammation, also host metabolism and takes part on the function of “gut - immune -nerve” axis [12]. Dysbiosis can cause the release of pro-inflammatory factors and disrupt the state of T cells, thereby affecting the peripheral nerve sensitivity and repair [13, 14]. The largest of the barrier microbiomes is also the skin microbiome and is important for maintaining local immune homeostasis and regulating inflammatory responses [15, 16]. Its dysregulation may alter the immune microenvironment around the median nerve, thereby participating in the pathogenesis of CTS. Although observational studies have suggested that these microbial factors may be linked to some immune or inflammatory mechanisms in CTS, there is no evidence for the actual cause.

Based on the above research background, the relationship between CTS and the control of the immune system, as well as the shift in the microbiome, may also be studied [17]. Previously, CTS was attributed to local mechanical factors; however, through observational studies, it has been suggested that systemic immune changes and low-grade inflammation may contribute to the onset and growth of CTS [18]. Additionally, as the role of the microbiome in modulating our body’s immunity and inflammation is gradually being accepted by people, the possible relation to our neurodegenerative disease has also turned into a new direction. However, the causal relationships among these factors with CTS, as well as the existence of an “immune-microecology-nerve” interactive pathway, are not supported by genetic evidence [19]. Therefore, in view of different aspects, such as immune, inflammation, and microbiota, and in combination with Mendelian Randomization (MR) to infer causality, it will be possible to gain a more systematic understanding of the pathogenesis of CTS and may also provide directions for future studies of possible therapeutic approaches.

MR, a method that uses genetic variants for causal inference, is able to lower the problem of confounding and reverse causation that are always present in traditional observational studies. In particular, the two-sample MR design leverages the genetic effects of exposures and outcomes from independent GWAS to get robust causal inference in different populations [20]. The two-sample MR method has been taken in the course of this research with the intention of studying the two-sample MR of peripherally immune cell subsets and an inflammatory protein’s effect on CTS risk. Furthermore, the features of the gut and skin microorganisms were introduced as mediators and were introduced by mediation MR analysis, constructing a causal path model of “immune / inflammation - microecology - CTS”, to elucidate the possible “immune - microecology - nerve” axis in CTS pathogenesis and provide a theoretical basis for targeted intervention.

## 2 METHODS

### 2.1 Study Design

The study design is visually presented in Fig. (**1**). To identify possible causal relationships among immune cells, inflammatory proteins, gut microbiota, skin microbiota, and CTS, we used the two-sample MR approach along with the two-step MR mediation. All analyses met STROBE-MR standards for methodological rigor and reliability [21, 22].

MR relies on three basic assumptions: first, the relevance assumption, that is the selected genetic markers are strongly related to the exposure; second, the independence assumption, which requires that the instrument is unrelated to potential confounders of the exposure-outcome relationship; Third, the exclusion restriction assumption, that is the genetic variants only affect the outcome through the exposure rather than through some other biological pathways [23]. These judgments about the presumptions were conducted *via* an instrumental variable selection process, and a bunch of sensitivity tests were also performed to examine diversity and horizontal pleiotropy.

### 2.2 Data Sources

Summary-level Genome-Wide Association Study (GWAS) data for CTS were obtained from the FinnGen R12 release (November 2024), comprising 31,181 CTS cases and 435,371 controls [24]. Immune cell trait data, including 731 phenotypes, were sourced from Chen *et al*. (sample size = 3,757) [GWAS IDs: GCST90001391–GCST90002121] [25]. Data on 91 inflammatory proteins came from Huang *et al*. (n = 14,824), based on Protein Quantitative Trait Loci (pQTL) analyses [GWAS IDs: GCST90274758–GCST902748^48^] [26]. Gut microbiota data were provided by Li *et al*., including 473 taxonomic units from 5,959 individuals, spanning 10 phyla, 18 classes, 24 orders, 58 families, 143 genera, 213 species, and 7 unclassified taxa [GWAS IDs: GCST90032172–GCST900326^44^] [27, 28]. Skin microbiota data originated from KORA FF4 and PopGen cohort studies (n = 597), profiled *via* 16S/ITS sequencing, with 150 taxa stratified by skin microenvironment types (dry, moist, sebaceous) [GWAS IDs: GCST90133164–GCST90133313] [29].

All GWAS data used were publicly available summary statistics from ethically approved original studies with participant consent. This study consisted solely of secondary analysis of public data and was exempt from additional ethical approval. It is worth noting that CTS cases and controls, as well as exposure datasets (immune cells, inflammatory proteins, microbiota), were from separate populations to minimize sample overlap and enhance robustness and validity.

### 2.3 SNP Selection and Instrumental Variable Validation

For this analysis, we selected immune SNPs, SNPs in inflammatory proteins, and SNPs near microbial taxa significantly associated with the traits of interest as IVs. In order to ensure that the selected SNPs had sufficient power and reliability, we used a genome-wide significance threshold and applied appropriate corrections according to the different genetic architectures and sample sizes of different phenotypes. For immune traits and inflammatory proteins, we used the standard genome-wide significance cutoff of *p* < 5×10^−8^, as this is widely used in GWAS studies [30]. This threshold avoided a large number of false positives due to recall of recurring test results and can guarantee that the selected SNPs are significantly correlated with the corresponding phenotype. This threshold is set to maximize statistical power to ensure that the SNPs reported in this paper are reliably associated with the traits of interest.

Due to the differences in genetic architecture and sample size, we relaxed significance thresholds for microbial taxa. For the skin microbiota, we used a *p* < 1×10^−5^ threshold because the genetic data of skin microbiota is relatively sparse and difficult. This threshold makes it more likely to capture the relevant SNPs [31]. For the gut microbiota, we used the *p* < 5×10^−6^ threshold, which was able to balance out the amount of power and the size of the samples, to make sure some important SNPs could be detected. These thresholds were set to achieve a balance between statistical power and sample size limitations, to ensure that the relevant SNPs were screened out without missing any potential associations [32]. To further ensure the independence of the selected SNPs, we did LD pruning at stringent cutoffs of r^2^ < 0.001 and > 10,000 kb. This step effectively reduced collinearity; each SNP represents an independent genetic variation, with no effect on the accuracy of the subsequent analysis [33]. Furthermore, to ensure the validity of the selected IVs, we calculated the F-statistics for each SNP and removed weak instruments with F-statistics less than 10 (Supplementary **1**). The F-statistic is used to verify whether the selected SNPs have sufficient power to explain the genetic association between exposures and outcomes, to form a stable basis for causal inference [34]. The corresponding SNP-exposure and SNP-outcome datasets were aligned to remove ambiguous palindromic SNPs (A/T, G/C) to eliminate strand alignment bias [35]. Bonferroni correction adjusted significance levels for multiple comparisons were as follows: 6.84×10^−5^ for immune cell subtypes (0.05/731); 5.49×10^−4^ for inflammatory protein expression levels (0.05/91); 1.05×10^−4^ for gut microbiota species abundance (0.05/473); 3.33×10^−4^ for skin microbiota species abundance (0.05/150). Associations having *p*-values falling between 0. 05 and the corrected threshold were regarded as suggestive. In order to reduce the influence of confounding factors, we selected SNP loci that were unrelated to potential confounders as instrumental variables. We then confirmed whether the selected SNPs showed significant correlations with possible confounders or other traits related to the study, including BMI, waist and hip circumference, body weight, smoking history, and education level.

We obtained key SNP information, such as chromosome position, Effect Allele (EA), Other Allele (OA), Effect Allele Frequency (EAF), effect size (β), Standard Error (SE), P-value, etc. The explained variance ratio (R^2^) and the F statistic were calculated as follows to assess instrument strength [36, 37]:

R^2^ = 2 × EAF × (1 - EAF) × β^2^ ÷ [2 × EAF × (1 - EAF) × β^2^ + 2 × EAF × (1 - EAF) × N × SE^2^];F = R^2^ × (N - 2) ÷ (1 - R^2^), where N is the effective sample size. The Odds Ratio (OR) derived from MR analyses quantified the causal effect of exposures on the binary CTS outcome [38].

### 2.4 MR Analysis

All statistics were carried out in R (version 4.4.1) using the MR packages, which comprise MRPRESSO (v1.0) and TwoSampleMR (v0.6.8). Five different MR methods were used, and the primary analysis used the inverse-variance-weighted method. Sensitivity analysis was carried out to test how estimates change when models are assumed to have different sensitivity analyses and to control for possible horizontal pleiotropy.

Heterogeneity was evaluated using Cochran’s Q test statistic; when *p* < 0.05, heterogeneity was significant. A MR-PRESSO global test and the MR-Egger intercept were used for horizontal pleiotropy; *p* > 0.05 implies no directional pleiotropy. Additionally, we performed leave-one-out analysis to investigate how individual SNPs affected the overall estimation.

### 2.5 Causal Mediation Analysis

Traits included in mediation analyses met the following criteria: (1) significant causal association with CTS without obvious heterogeneity or pleiotropy; (2) consistent instrument selection criteria applied to assess effects of immune/inflammatory traits on candidate mediators; (3) two-step MR was implemented to estimate total effect (β_all) of immune/inflammatory exposure on CTS, effect on mediator (β_1_), and mediator effect on CTS (β_2_). The mediation effect was calculated by the product of the coefficients (β_1_ × β_2_), with the mediation proportion estimated as (β_1_ × β_2_) / β_all. The 95% Confidence Interval (CI) of mediation effects was computed using the Delta method [39]. Direct effects were obtained by subtracting the mediation effect from the total effect (β_all – β_1_ × β_2_). Mediation *p* < 0.05 indicated statistical significance.

### 2.6 Reverse MR Analysis

In reverse MR, CTS was the exposure, and immune cells, inflammatory proteins, gut and skin microbiota were outcomes. SNPs significantly associated with CTS (*p* < 5 × 10^−6^) were used as the IVs, and Bonferroni-corrected significance thresholds were 6.84 × 10^−5^ (immune cells), 5.49 × 10^−4^ (inflammatory proteins), 1.05 × 10^−4^ (gut microbiota), and 3.33 × 10^−4^ (skin microbiota). *p* values from 0.05 to corrected thresholds were regarded as suggestive.

## 3 RESULTS

Statistical significance is mainly based on the IVW method. An analysis was carried out in a systematic order: first, the causality of immune cell phenotype and inflammatory proteins on CTS was evaluated, and then the causality of gut and skin microbiota on CTS was evaluated. Next, we carried out reverse MR to determine whether CTS could affect immune cell phenotypes, inflammatory proteins, and gut and skin microbiota. Then, we examined whether immune cells and inflammatory proteins were affecting the gut and skin microbiota. Finally, we computed the mediation effects, and associations were deemed statistically meaningful only if the IVW *p*-value was less than the Bonferroni-corrected threshold. All the results that met this criterion were presented in the corresponding forest plots.

### 3.1 Causal Relationships Between Immune Cell Traits and CTS

**Fig. (2):**
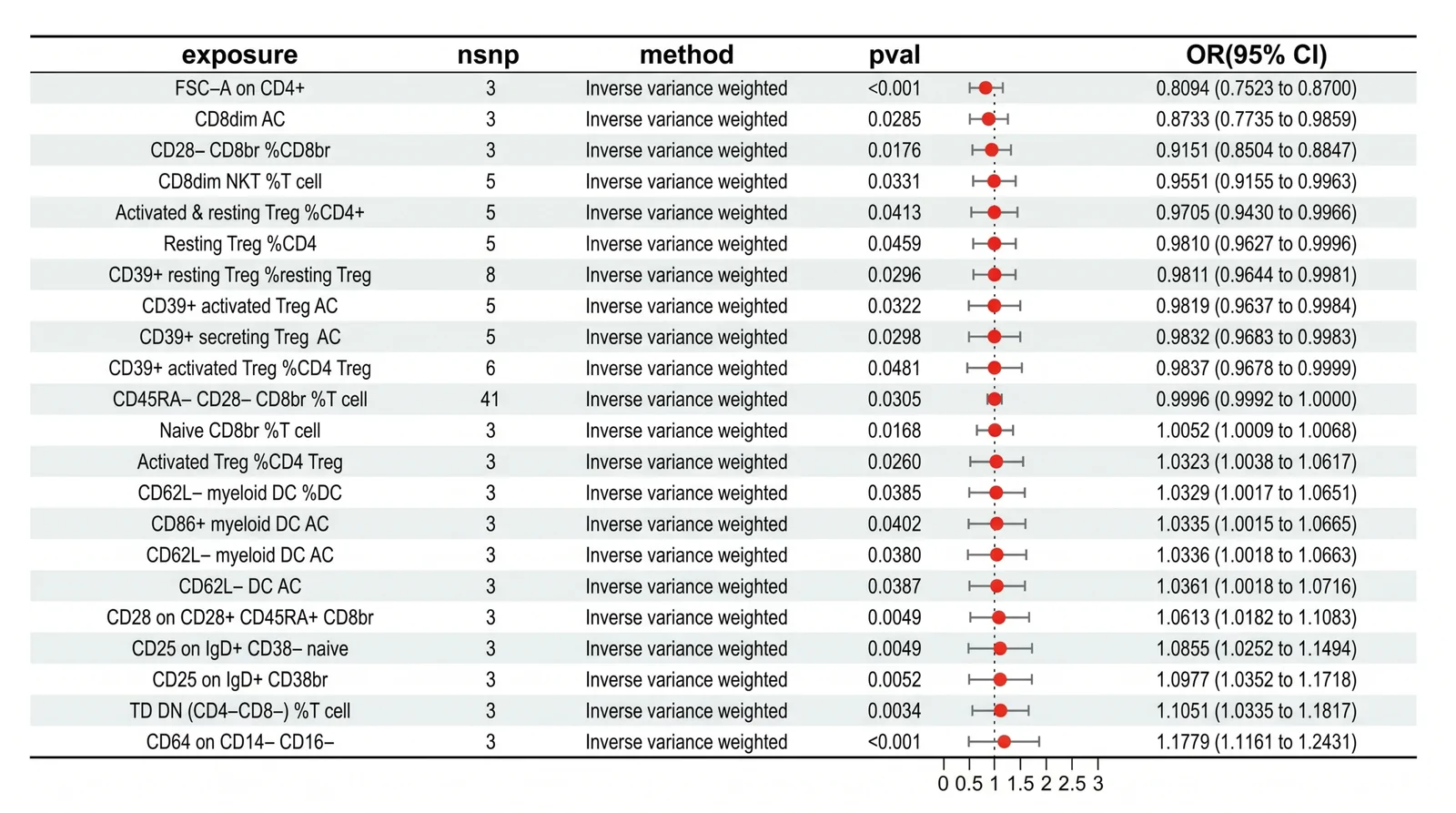
MR forest plot of immune cell traits and CTS.

Two-sample MR analysis identified 22 immune cell phenotypes that showed causal associations with CTS, including 11 protective traits and 11 traits associated with increased risk. Specifically, traits such as FSC−A on CD4^+^ (OR = 0.8094, 95% CI: 0.7523–0.8708), CD8dim AC (OR = 0.8733, 95% CI: 0.7735–0.9859), CD28^−^ CD8br %CD8br (OR = 0.9151, 95% CI: 0.8504–0.9847), and CD8dim NKT %T cell (OR = 0.9551, 95% CI: 0.9155–0.9963) showed protective effects. In contrast, increased expression of CD64 on CD14^−^ CD16^−^ (OR = 1.1779, 95% CI: 1.1161–1.2431), TD DN (CD4^−^CD8^−^) %T cell (OR = 1.1051, 95% CI: 1.0335–1.1817), CD25 on IgD^+^ CD38br (OR = 1.0977, 95% CI: 1.0283–1.1718), and CD25 on IgD^+^ CD38^−^ naive (OR = 1.0855, 95% CI: 1.0252–1.1494) were associated with increased CTS risk (Fig. **2**). In addition, results were consistent across the other four MR methods (Supplementary Fig. **1**), and sensitivity analyses further confirmed the robustness of the findings. No significant heterogeneity or horizontal pleiotropy was detected (Supplementary Tables **1** and **2**).

### 3.2 Causal Relationships Between Inflammatory Proteins and CTS

**Fig. (3):**
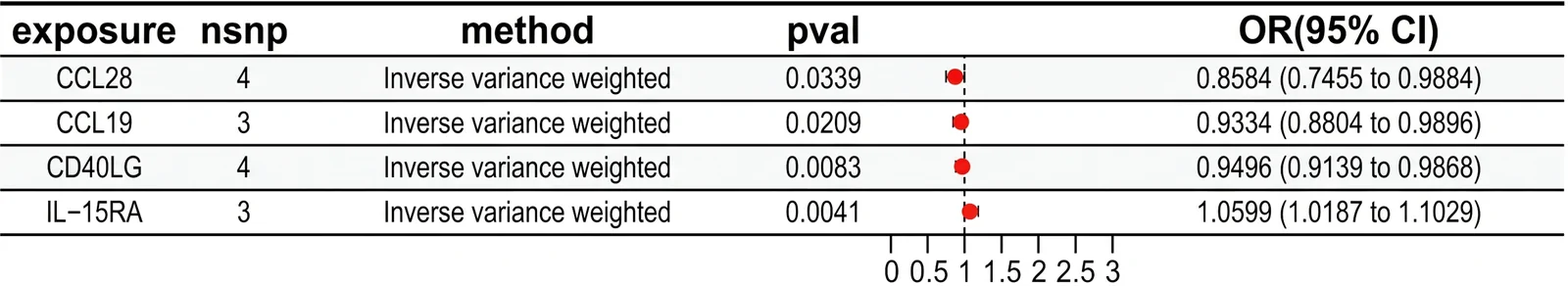
MR forest plot of inflammatory proteins and CTS.

Two-sample MR analysis identified four inflammatory proteins causally associated with CTS, including three with protective effects and one associated with increased risk. Specifically, CCL28 (OR = 0.8584, 95% CI: 0.7455–0.9884), CCL19 (OR = 0.9334, 95% CI: 0.8804–0.9896), and CD40LG (OR = 0.9496, 95% CI: 0.9139–0.9868) showed protective effects, whereas IL-15RA (OR = 1.0599, 95% CI: 1.0187–1.1029) was associated with increased CTS risk (Fig. **3**). Consistent results were observed across the other four MR methods (Supplementary Fig. **2**), and sensitivity analyses supported the robustness of the results. No significant heterogeneity or horizontal pleiotropy was observed (Supplementary Tables **3** and **4**).

### 3.3 Causal Relationships Between Gut Microbiota and CTS

**Fig. (4):**
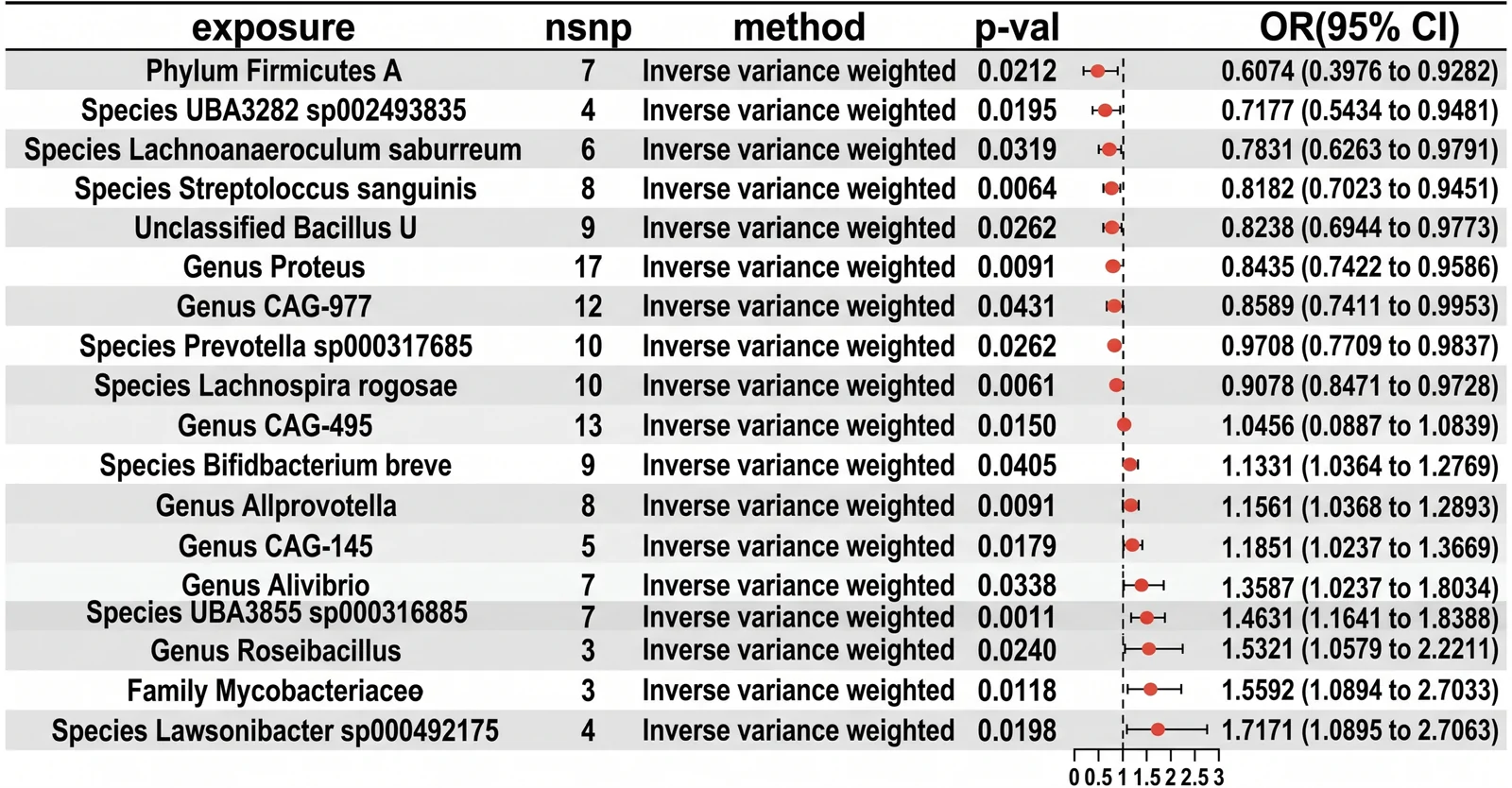
MR forest plot of gut microbiota and CTS.

Two-sample MR analysis identified 18 gut microbial taxa that were significantly causally associated with CTS, including nine taxa with protective effects and nine associated with increased risk (including one phylum, one family, eight genera, eight species, and one unclassified taxon). Specifically, certain taxa showed a strong inverse association with CTS risk. For example, *Phylum Firmicutes A* (OR = 0.6074, 95% CI: 0.3976–0.9282) demonstrated a marked protective effect. *Species* such as *UBA3282 sp002493835* (OR = 0.7177, 95% CI: 0.5434–0.9481), *Lachnoanaerobaculum saburreum* (OR = 0.7831, 95% CI: 0.6263–0.9791), and *Streptococcus sanguinis* (OR = 0.8182, 95% CI: 0.7083–0.9451) also exhibited protective associations. On the other hand, several taxa were positively associated with CTS risk. Notably, *Species Lawsonibacter sp000492175* (OR = 1.7171, 95% CI: 1.0895–2.7063) and *Family Mycobacteriaceae* (OR = 1.5592, 95% CI: 1.1034–2.2033) showed relatively high OR values, suggesting their elevated abundance may significantly increase CTS risk. Other taxa, such as *Genus Roseibacillus* (OR = 1.5329, 95% CI: 1.0579–2.2211) and *Species UBA3855 sp900316885* (OR = 1.4631, 95% CI: 1.1641–1.8388) also showed positive associations, albeit with milder effects (Fig. **4**). Results from the other four MR methods were consistent (Supplementary Fig. **3**), and sensitivity analyses further supported the robustness of these findings. No evidence of significant heterogeneity or horizontal pleiotropy was detected (Supplementary Tables **5** and **6**).

### 3.4 Causal Relationships Between Skin Microbiota and CTS

**Fig. (5):**
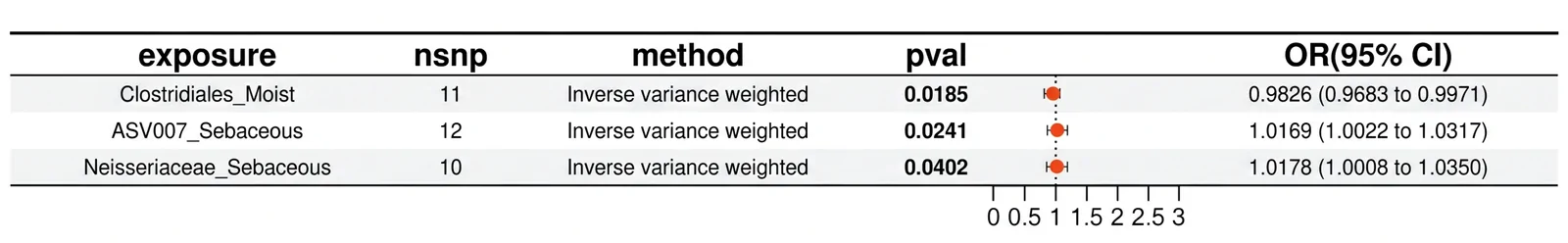
MR forest plot of skin microbiota and CTS.

Two-sample MR analysis identified three skin microbial taxa with causal associations with CTS, including one with a protective effect and two associated with increased risk. Specifically, *Clostridiales_Moist* (OR = 0.9826, 95% CI: 0.9683–0.9971) showed a protective effect, whereas *ASV007_Sebaceous* (OR = 1.0169, 95% CI: 1.0022–1.0317) and *Neisseriaceae_Sebaceous* (OR = 1.0178, 95% CI: 1.0008–1.0350) were associated with increased CTS risk (Fig. **5**). These results were consistent across the other four MR methods (Supplementary Fig. **4**), and sensitivity analyses confirmed the robustness of the findings. No substantial heterogeneity or horizontal pleiotropy was observed (Supplementary Tables **7** and **8**).

### 3.5 Reverse MR Analysis

To evaluate the directionality of causal associations, reverse MR analysis was conducted to examine whether genetic susceptibility to CTS influences the composition of immune cell phenotypes, inflammatory proteins, gut microbiota, and skin microbiota. The analysis revealed significant causal effects of CTS on the following immune cell phenotypes: CD39^+^ activated Treg %CD4 Treg, CD39^+^ secreting Treg AC, and CD39^+^ resting Treg %resting Treg. No significant associations were observed for the other 19 immune cell traits.

Further reverse MR analyses revealed significant causal effects of CTS on the gut microbial taxa *Genus CAG−145*, *Genus CAG−977*, *Phylum Firmicutes A*, and *Unclassified Bacillus U*. No significant associations were observed for the remaining 14 taxa.

**Fig. (6):**
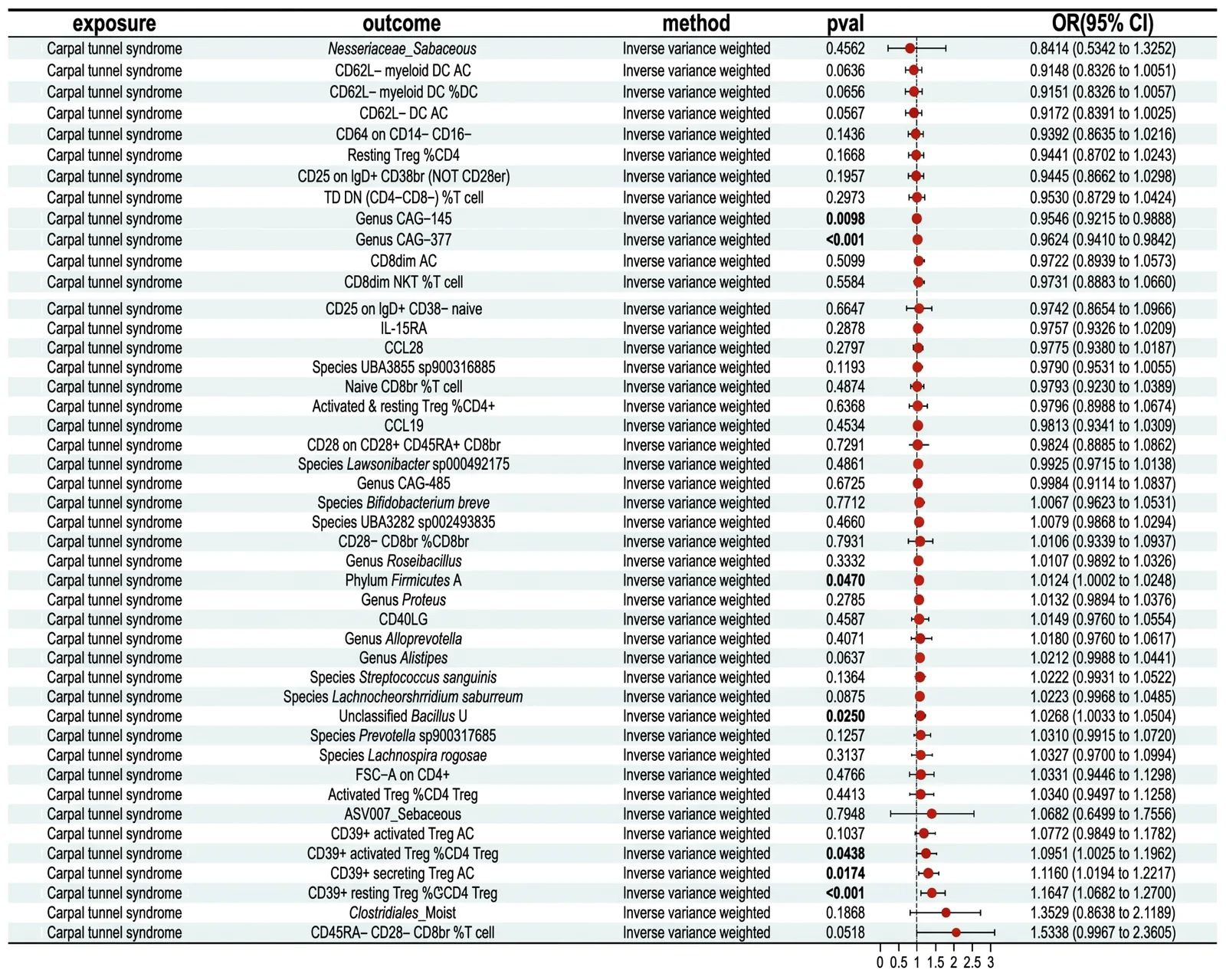
Mendelian randomization results presented as forest plots.

In addition, no significant causal associations were observed between CTS and the four inflammatory proteins or the three skin microbial taxa (Fig. **6**).

### 3.6 Mediation Analysis

**Fig. (7):**
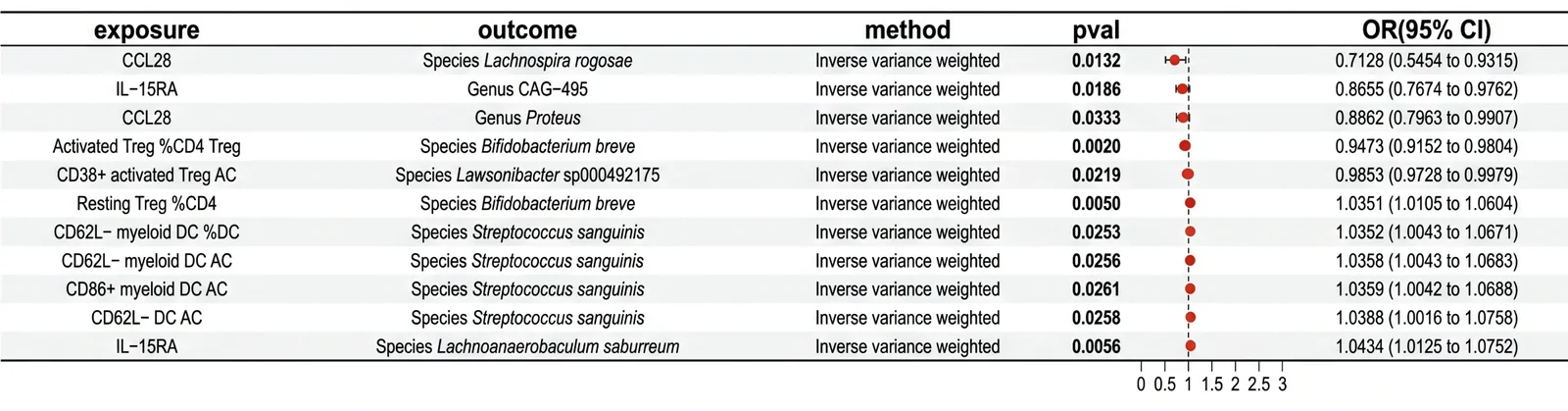
Mediation analysis of CTS mediated by gut or skin microbiota through immune cells or inflammatory proteins.

To enhance the validity of causal inference, taxa that display reverse causality were excluded from the mediation analysis. The final analysis included 19 immune cell phenotypes and 4 inflammatory proteins (the exposures), and 14 gut microbial taxa and 3 skin microbial taxa (the outcomes). Using a two-step MR approach, we have examined whether there are any mediating effects systematically. According to the preset screening criteria, seven microbial taxa (two genera and five species) were identified as potential mediators in the causal pathways from immune or inflammatory factors to CTS (Fig. **7**). For example, CCL28 was significantly negatively correlated with the species Lachnospira rogosae (OR = 0.7128, 95% CI: 0.5454 - 0.9316). Subsequent analysis also showed that some microbial taxa were regulated by multiple immune phenotypes. *Species Streptococcus sanguinis* was positively regulated by the following Dendritic Cell (DC)-related phenotypes: CD62L^−^ myeloid DC %DC (OR = 1.0352, 95% CI: 1.0043–1.0671), CD62L^−^ myeloid DC AC (OR = 1.0358, 95% CI: 1.0043–1.0683), CD86^+^ myeloid DC AC (OR = 1.0359, 95% CI: 1.0042–1.0686), and CD62L^−^ DC AC (OR = 1.0386, 95% CI: 1.0046–1.0738). In addition, *Species Bifidobacterium breve* was inhibited by Activated Treg %CD4 Treg (OR = 0.9473, 95% CI: 0.9153 - 0.9804) and promoted by Resting Treg %CD4 (OR = 1.0351, 95% CI: 1.0105 - 1.0604). Sensitivity analyses with heterogeneity and horizontal pleiotropy evaluations were undertaken, and also provide support for our findings (Supplementary Tables **9** and **10**).

To further investigate whether there are mediating effects of gut and skin microbiota in the paths between immune cell phenotypes and inflammatory protein and CTS, a two-step MR mediation analysis was conducted (Table **1**). A few microbial taxa showed statistically significant mediation effects. For example, *Species Lawsonibacter sp000492175* partially played a role in the effects of CD39^+^activated Treg AC on CTS, with a mediation effect estimated at -0.00802 (95%CI: -0.0149, -0.00116), accounting for 44.0% of the total effect (95% CI: 6.36% - 81.7%, *p* = 0.0219). Likewise, 41.1% of the effect of activated Treg %CD4 Treg on CTS was mediated by *Species Bifidobacterium breve* (95% CI: 12.7% to 69.5%), and the mediation effect was -0.00676 (95% CI: -0.0114 to -0.00209, *p* = 0.0046).

Interestingly, the other was Bifidobacterium breve, with an opposite-direction mediation effect between resting Treg %CD4 and CTS, which may be a “suppressor effect” with a mediation effect of 0.00431 (95% CI: 0.00119-0.00744) and accounts for –22.5% (95% CI: –38.8% to –6.19%, *p* = 0.0069). Furthermore, *Species Streptococcus sanguinis* was repeatedly identified as a mediator in four DC-related immune phenotypes: CD62L^−^ myeloid DC %DC, CD62L^−^ myeloid DC AC, CD62L^−^ DC AC, and CD86^+^ myeloid DC AC, and the mediation effect was in the range of -0.00694 to -0.00760, with a consistent mediation proportion of about -21.4% to -21.5%, all of which were statistically significant *p* value (0.0275 to 0.0285). In addition, IL-15RA also exerted a partial effect of mediation on CTS through Lachnoanaerobaculum saburreum; the mediation effect was -0.0104 (95% CI: -0.0178 to -0.00292), and the mediation rate was –17.8% (95% CI: –30.7% to –5.02%, *p* = 0.0064).

It is also worth noting that the skin microbiota cannot be considered as an important mediator among immune cell phenotype, inflammation protein, and CTS due to a lack of evidence of a considerable mediatory effect. Specific gut microbial taxa, such as *Species Bifidobacterium breve*, *Species Streptococcus sanguinis*, and *Species Lawsonibacter sp000492175*, together can play a role as mediators in immune regulation and CTS pathogenesis. Some pathways have been observed to have suppressor effects, indicating that there is a multi-pathway regulation in the underlying mechanism of CTS.

## 4 DISCUSSION

This study systematically applied the two-sample MR and two-step mediation MR methods to study the causal effect and potential mediating effect of immune cell phenotype, inflammatory protein, gut microbiota, and skin microbiota in the pathogenesis of CTS. Thus, some immune cell phenotypes and inflammatory proteins are not only causally linked to CTS on their own but can also influence CTS development *via* some of the taxa within the gut microbiome. Skin microbiota was linked to CTS, but no evidence was found to suggest this mediated *via* an immune/inflammatory pathway in our model, so it appears that skin microbiota may not have been a large mediator in this case. Moreover, these results also provide a new understanding of the immune-microecological regulatory mechanism of CTS and provide a theoretical basis for targeted therapy of CTS.

### 4.1 Immune Cell Phenotypes and CTS: New Evidence of Neuro-immune Crosstalk

We identified 22 immune cell phenotypes that were significantly causally associated with CTS, suggesting that the peripheral immune system is likely a contributing factor. Several CD8^+^T cell subsets (CD8dimAC, CD28-CD8br %CD8br, etc.) have protective effects, which can be involved in regulating peripheral nerve inflammation. Previous research has shown that CD8^+^ T cells participate in nerve repair by secreting IL-10 and other factors to minimize neuroinflammation damage [40]. On the contrary, the activation-related phenotypes, including CD25^+^IgD^+^B cells, correlated with the upregulation of CTS risk, implying that T/B cell activation may induce local inflammation and exacerbate median nerve compression. A subset such as Dendritic Cell (DC) with CD62L-myeloid DC%DC, was found to have a very strong correlation with the protective gut microbe population, suggesting that DC-mediated antigen presentation could be at the heart of regulation by the immune-microecology system [41].

### 4.2 Dual Roles of Inflammatory Proteins: Regulatory versus Pro-inflammatory Chemokines

This study is the first to report a negative causal relationship between CCL28, CCL19, and CD40LG with CTS, and elevated IL-15RA expression more than doubled the risk of CTS. CCL28 attracts Treg cells to the inflammatory site, maintains tissue immune tolerance, and may suppress chronic CTS inflammation response [42]. CCL19, which helps lymphocyte homing and clearance in chronic inflammation tissue, such as synovial tissues, generally indicates that inflammation is on the decline [43]. The protective effect of CD40LG may be through its induction of anti-inflammatory M macrophage polarization and tissue repair [44]. However, in IL-15RA, the primary activator of CD8^+^ T cells and NK cells, prolonged inflammation can also be observed in autoimmunity like RA [45], which could exacerbate CTS *via* further upregulating the recruitment of immune cells and increasing secretion of cytokines, worsening local edema and nerve compression.

### 4.3 Gut Microbiota and CTS: Microbial Signals as Nodes of Inflammatory Regulation

Eighteen gut microbial taxa were causally linked to CTS; nine of these showed protective effects. Several Firmicutes taxa, such as *Species Streptococcus sanguinis*, *Species Lachnoanaerobaculum saburreum*, and *Species UBA3282 sp002493835*, are related to Short-Chain Fatty Acid (SCFA) production Immune homeostasis [46]. *Species Streptococcus sanguinis* induces TGF-β expression and promotes an anti-inflammatory response, which may be of use to patients with CTS, *i.e.*, patients with chronic inflammation and nerve compression [47]. On the other hand, there is a high abundance of the *species Lawsonibacter sp000492175* and the *family Mycobacteriaceae*, which are associated with a higher risk of CTS. It may be induced by the Lipopolysaccharide (LPS)-NF-κB pathway, which is able to amplify inflammatory responses [48]. The observation that many different microbes are being turned on or off by different immune phenotypes indicates that maybe gut microbes could be doing more than just being downstream results; they may also serve as important feedback nodes in immune regulatory networks.

### 4.4 Skin Microbiota and CTS: Marginal Participation or Potential Modulators

Three skin microbial taxa were found to be causally linked to CTS. A higher abundance of *Clostridiales_Moist* is associated with a lower risk of CTS, suggesting an immunomodulatory effect in the skin mucosal barrier. Earlier studies show that Clostridiales can lead to the proliferation of skin Tregs and regulate the response of keratinocytes to inflammation [49]. While *Neisseriaceae* and *ASV007* (enriched in the sebaceous skin site) were positively correlated with CTS risk (induce local sebaceous gland inflammation, upregulate IL-1β, mediate distal immune effect [50]. Even though the causal effect was weak, these results do suggest that the skin microbiota can affect CTS *via* a “skin-peripheral nerve” pathway and warrant further mechanistic research.

### 4.5 Reverse Mendelian Randomization Analysis: CTS as a Potential Driver of Immune and Microbial Remodeling

In addition to the forward causal effects, reverse MR analysis showed that CTS also has important impacts on various immune characteristics and gut microbiota features, indicating that CTS itself may initiate and drive the regulation of the whole-system immune response and microbial community reconstruction. As a result, CTS produced reverse effects on *Genus CAG−145*, *Genus CAG−977*, *Phylum Firmicutes A*, *Unclassified Bacillus U*, and some CD39^+^ regulatory T cells (Treg)-related phenotypes such as CD39^+^ activated Treg %CD4 Treg, CD39^+^ secreting Treg AC, and CD39^+^ resting Treg %resting Treg.

These conclusions provide evidence that CTS is not merely a passive result of dysregulation of the immune system or microorganisms, but rather an active regulator that causes a series of subsequent immune regulatory processes. The pathological process of CTS is usually characterized by a chronic nerve compression state, local ischemia, and a state of long-term low-grade inflammation, which may lead to continuous immune activation and subsequently trigger the process of compensatory immunoregulation. CD39^+^ Tregs play an important role in maintaining immune homeostasis through the exogenous ATP into adenosine [51]. The changes observed across the different CD39^+^ Treg subsets may therefore represent an adaptive immune regulatory response to CTS-related inflammatory stress, to constrain excessive immune activation and tissue injury.

In addition, the reverse effects of CTS on specific gut microbial taxa, including Firmicutes A and the poorly understood CAG−145 and CAG−977, indicate the possible existence of a “gut–peripheral nerve axis”. CTS-induced chronic pain, inflammation, and dysfunction could influence the systemic metabolic state and neuroimmune signaling pathways, which in turn reshape the gut microbe profile. Although the biological functions of some taxa are still partially unexplored, prior studies have shown that modifications in Firmicutes-related taxa can be associated with an increased inflammatory burden and immune modulation, which offers some indirect evidence for the involvement of these taxa in the development of systemic effects in CTS.

More importantly, it can be observed from the reverse MR analysis that there may also be a bidirectional relationship between CTS and immune - microbial feature, as we know that chronic peripheral nerves were mostly more like a result that neuropathy induces systemic immune remodeling, rather than a disorder secondary to preexisting systemic immune disease [52]. Nevertheless, these results should be interpreted with caution as reverse MR reflects genetically predicted susceptibility to CTS and will not fully capture the severity or duration of the disease, or whether it is treatment-related.

Overall, the reverse associations observed in this study suggest that CTS may promote the onset and development of disease by dynamically regulating the immune system and the composition of the gut microbiota. Future longitudinal and experimental studies should confirm these findings and explain the sequence of the timepoints and molecular mechanisms by which CTS affects the immune system and microorganisms.

### 4.6 Microecological Mediation Mechanisms: Unveiling Pathway Diversity and Directionality

Two-step MR mediation analyses showed a number of microbial species as mediators of immune/inflammatory factors in CTS. *Species Bifidobacterium breve* mediated 41.1% of the effect of activated Treg % CD4 Treg on CTS, which may lead to the Foxp3^+^ Treg expansion, to modify the gut barrier function, and to reduce the pro-inflammation pathway [53, 54]. Interestingly, we see that the protective elevation of resting Treg (%CD4) levels normally exerts some of those benefits by increasing the abundance of Bifidobacterium breve. These species then have an effect on CTS. However, this bacterium is positively correlated with CTS development, showing a reverse mediation effect. This means that in the specific immune microenvironment of CTS, B. breve might reframe the beneficial messages conveyed by Treg cells, leading them towards a microbial state that exacerbates the disease, and to demonstrate that the bacterium has highly context-specific functional characteristics [55]. On the other hand, the activation status of Dendritic Cells (DCs) is closely related to chronic inflammation, and under the condition of partial direct injury, the enrichment of the *species Streptococcus sanguinis* can partially mediate this influence. This bacterium consistently showed negative regulatory effects on four DC-related immune phenotypes and was finally associated with the improvement or resolution of CTS. Thus, S. sanguinis, the microbiome is playing a key “signal regulator” role: S. sanguinis is a functional “suppressor” with respect to CTS, since it is present, it is induced by harmful upstream immune states, and it actively diminishes and reverses the effect on CTS that would otherwise be induced by the upstream immune state. This is not due to the bacterium's direct suppression of immune cells but rather by the bacterium mediating the 'immune exposure → microbiota change → disease outcome' process to exert a negative regulation and ultimately 'correcting' the immune dysregulation signal, and exerting a protective effect on the disease. In this study, we found that Lawsonibacter sp000492175 has an important mediating effect between CD39^+^ Treg cell activation and CTS, and the mediating effect ratio can reach 44%. This indicates that CD39^+^ Treg cells may regulate Lawsonibacter sp000492175 to influence the immune response and thus participate in the immune pathology of CTS. CD39^+^ Treg cells may hydrolyze extracellular ATP to produce immunosuppressive adenosine and inhibit excessive immune responses. As a kind of gut microbiota, the abundance of Lawsonibacter sp000492175 may be regulated by CD39^+^Treg cells, thereby affecting immune homeostasis and changing the pattern of immune responses. The immunosuppressive effect of CD39^+^ Treg cells may have an important role in the composition of the gut microbiota and the body’s immune response to Lawsonibacter sp000492175, which then influences the function of the latter and controls the immune pathogenesis of CTS [56]. IL-15RA may partially mediate effects *via* *Species Lachnoanaerobaculum saburreum*, which could promote acetate production and influence mucosal immune barrier regulation, suggesting a potential link between metabolism and immune function in CTS pathogenesis [57]. It is not that the skin microbiota lacks a mediating effect, but rather that it shows no significant mediation effect, indicating that the skin microbiota may not play a significant role in the systemic immune regulation mechanism of CTS. The skin microbiota mainly affects the local immune response, especially at the site of infection or injury; its disruption alters the immune environment at the site of median nerve compression [58]. However, in the context of a systemic disease such as CTS with peripheral nerve inflammation and an immune disorder, the skin microbiota may not be able to effectively mediate the influence of the immune system on a larger scale. Although our study did not find a mediating effect of skin microbiota, this result may be due to the lack of data and statistical power. There is relatively little data on the skin microbiota, and its impact on the immune system is mostly localized immune modulation rather than systemic immune regulation. Therefore, although the skin microbiota can participate in local immune responses, it does not appear to mediate the systemic immune regulation of CTS, and it may not be the primary mediator of this disease.

On the contrary, gut microbiota emerged as an important factor in the relationship between immune cell phenotype and CTS, indicating that it plays a key role in regulating the immune system. Unlike Skin microbiota, which only affects local immunity, gut microbiota, which has a systemic effect, influences the human body's immune system. The gut microbiota regulates the function and state of immune cells through interactions with immune cells and produces metabolites such as SCFAs [59]. These mechanisms play an important role in regulating peripheral nerve sensitivity and repair. They also include immune cells like T cells and inflammatory mediators, which will have an impact on gut microbes by promoting dysbiosis or changing the microbial diversity [60]. Dysbiosis of the gut microbiota then interferes with the differentiation and function of immune cells, and this worsens inflammation in peripheral nerves and the course of CTS.

In addition, inflammatory cytokines such as TNF-α and IL-6, which are elevated in CTS, can promote the growth of pro-inflammatory bacteria and the damage of beneficial ones in the gut microbiota [61]. This dysbiosis may further promote the systemic inflammation and immune dysfunction of CTS. In light of how immune cells, inflammation, and gut microbiota interact, the immune/inflammation-gut-nerve axis shows the pathogenesis of CTS.

### 4.7 Limitations and Future Directions

This study is the first causal inference study to combine immune and microecological factors in the pathogenesis of CTS, and it has proposed a dual-pathway regulatory model of “immune-microecology”. The results provide a theoretical basis for microbiome- or immune-targeted interventions for CTS. The GWAS datasets predominantly consist of individuals of European ancestry, which may limit the generalizability of our findings to other populations. Moreover, the detection of the existence of the mediation effect is based on the magnitude of the effect, which means that it is possible that some weak mediating processes will be missed. Although we carried out pleiotropy testing in the analysis to reduce the impact of horizontal pleiotropy on the results, the problem of unobserved pleiotropic effects remains, especially for some SNPs that affect multiple traits at the same time. Future work needs to use multi-omics, longitudinal cohort, and experimental methods to demonstrate the biological feasibility of the causal pathways mentioned above and find out “high-risk” microbial labels for CTS risk assessment and early intervention.

## CONCLUSION

By using the combination of two-sample MR and mediation analysis, this paper shows that immune cell phenotypes and inflammatory proteins may influence the risk of CTS *via* specific gut microbiota taxa. *Species Streptococcus sanguinis* and *Species Bifidobacterium breve* were found to be able to transmit immune or inflammatory signals to the development of CTS. Skin microbiota was associated with CTS, but no evidence for a mediation effect was observed. In general, this research provides a theoretical basis for further research and understanding of the mechanism involved in the immune-microecological interaction in the pathogenesis of CTS and provides certain references and directions to follow.

## Figures and Tables

**Fig. (1):**
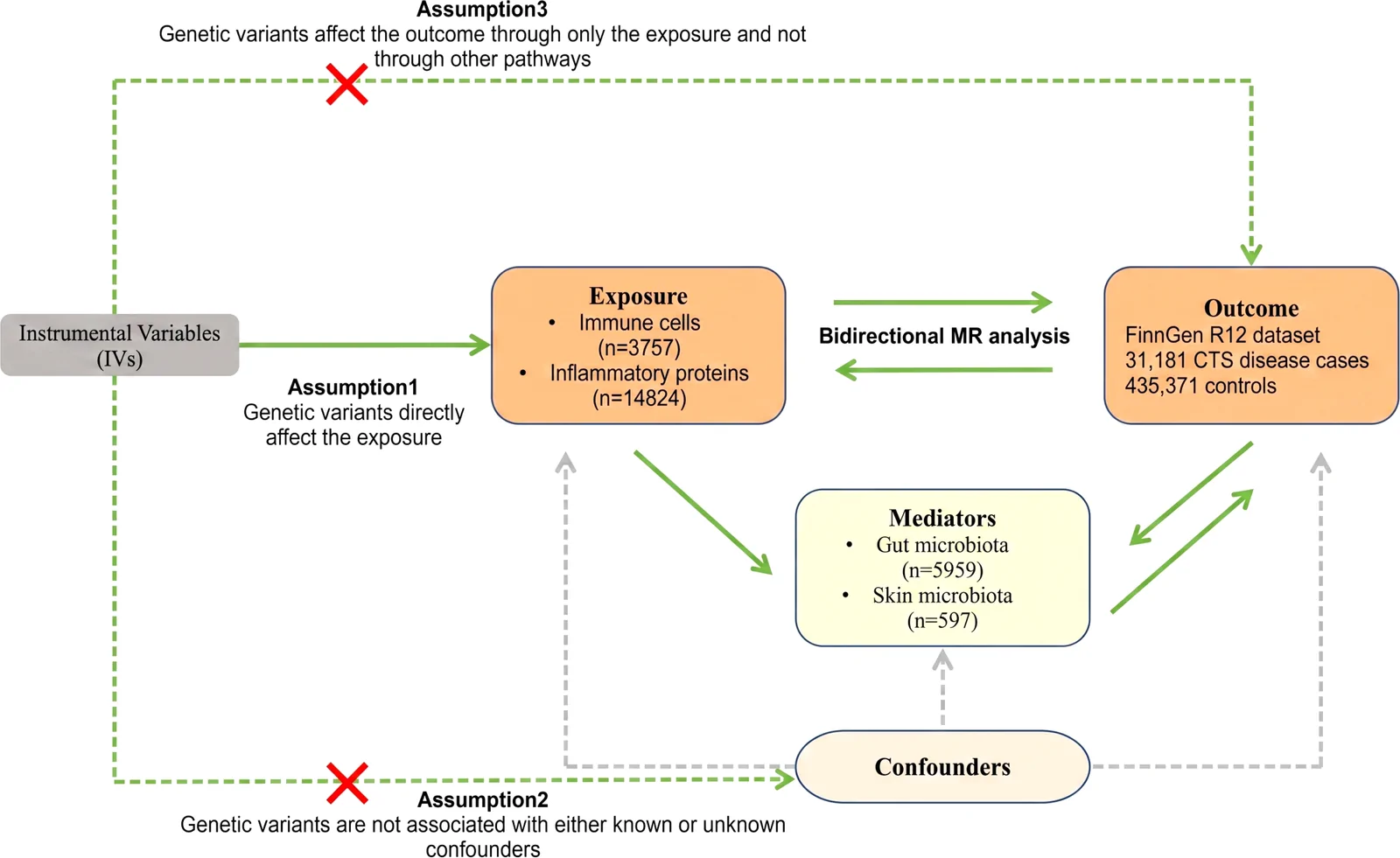
Flow chart of the MR study.

**Table 1:** Mediation effects with proportion estimates for each candidate mediator.

**Exposures**	**Mediator**	**Outcome**	**Mediated Effect**	**Mediated** **Proportion**	**Direct Effect**	***p*-value**
CD39^+^ activated Treg AC	*Species Lawsonibacter sp000492175*	CTS	-0.00802 (-0.0149, -0.00116)	44% (6.36%,81.7%)	-0.010206137	0.021945095
Activated Treg %CD4 Treg	*Species Bifidobacterium breve*	CTS	-0.00676 (-0.0114, -0.00209)	41.1% (12.7%,69.5%)	-0.009689805	0.004601647
Resting Treg %CD4	*Species Bifidobacterium breve*	CTS	0.00431 (0.00119, 0.00744)	-22.5% (-6.19%, -38.8%)	-0.023482876	0.00685723
CD62L- myeloid DC %DC	*Species Streptococcus sanguinis*	CTS	-0.00694 (-0.0131, -0.000768)	-21.4% (-40.5%, -2.37%)	0.039331576	0.027530604
CD62L- myeloid DC AC	*Species Streptococcus sanguinis*	CTS	-0.00706 (-0.0134, -0.000772)	-21.4% (-40.4%, -2.34%)	0.0400894	0.027777238
CD62L- DC AC	*Species Streptococcus sanguinis*	CTS	-0.0076 (-0.0144, -0.000801)	-21.4% (-40.6%, -2.26%)	0.043108891	0.028449828
CD86+ myeloid DC AC	*Species Streptococcus sanguinis*	CTS	-0.00707 (-0.0134, -0.000745)	-21.5% (-40.7%, -2.26%)	0.040014453	0.02847939
IL-15RA	*Species Lachnoanaerobaculum saburreum*	CTS	-0.0104 (-0.0178, -0.00292)	-17.8% (-30.7%, -5.02%)	0.068605	0.006369

## Data Availability

The data that support the findings of this study are available in the supplementary material of this article.

## LIST OF ABBREVIATIONS

CTS: Carpal Tunnel Syndrome
pQTL: Protein Quantitative Trait Loci
CRP: C Reactive Protein
LD: Linkage Disequilibrium
SCFA: Short Chain Fatty Acid
MR: Mendelian Randomization
IVW: Inverse Variance Weighted
IVs: Instrumental Variables
GWAS: Genome Wide Association Studies
OR: Odds Ratio
β: Effect Sizes
CI: Confidence Interval

## STANDARDS OF REPORTING

STROBE-MR guidelines were followed.
